# A Reduction in Adult Blood Stream Infection and Case Fatality at a Large African Hospital following Antiretroviral Therapy Roll-Out

**DOI:** 10.1371/journal.pone.0092226

**Published:** 2014-03-18

**Authors:** Nicholas A. Feasey, Angela Houston, Mavuto Mukaka, Dan Komrower, Thandie Mwalukomo, Lyson Tenthani, Andreas Jahn, Mike Moore, Remco P. H. Peters, Melita A. Gordon, Dean B. Everett, Neil French, Joep J. van Oosterhout, Theresa J. Allain, Robert S. Heyderman

**Affiliations:** 1 Malawi Liverpool Wellcome Trust Clinical Research Programme (MLW), University of Malawi College of Medicine, Blantyre, Malawi; 2 Department of Medicine, University of Malawi College of Medicine, Blantyre, Malawi; 3 Liverpool School of Tropical Medicine, Liverpool, United Kingdom; 4 Department of HIV and AIDS, Ministry of Health, Lilongwe, Malawi; 5 I-TECH Lilongwe, Malawi; 6 I-TECH, Department for Global Health, University of Washington, Seattle, Washington, United States of America; 7 Anova Health Institute, Johannesburg and Tzaneen, South Africa; 8 Institute of Infection and Global Health, University of Liverpool, Liverpool, United Kingdom; 9 Dignitas International, Zomba, Blantyre, Malawi; University of New South Wales, Australia

## Abstract

**Introduction:**

Blood-stream infection (BSI) is one of the principle determinants of the morbidity and mortality associated with advanced HIV infection, especially in sub-Saharan Africa. Over the last 10 years, there has been rapid roll-out of anti-retroviral therapy (ART) and cotrimoxazole prophylactic therapy (CPT) in many high HIV prevalence African countries.

**Methods:**

A prospective cohort of adults with suspected BSI presenting to Queen's Hospital, Malawi was recruited between 2009 and 2010 to describe causes of and outcomes from BSI. Comparison was made with a cohort pre-dating ART roll-out to investigate whether and how ART and CPT have affected BSI. Malawian census and Ministry of Health ART data were used to estimate minimum incidence of BSI in Blantyre district.

**Results:**

2,007 patients were recruited, 90% were HIV infected. Since 1997/8, culture-confirmed BSI has fallen from 16% of suspected cases to 10% (p<0.001) and case fatality rate from confirmed BSI has fallen from 40% to 14% (p<0.001). Minimum incidence of BSI was estimated at 0.03/1000 years in HIV uninfected vs. 2.16/1000 years in HIV infected adults. Compared to HIV seronegative patients, the estimated incidence rate-ratio for BSI was 80 (95% CI:46–139) in HIV-infected/untreated adults, 568 (95% CI:302–1069) during the first 3 months of ART and 30 (95% CI:16–59) after 3 months of ART.

**Conclusions:**

Following ART roll-out, the incidence of BSI has fallen and clinical outcomes have improved markedly. Nonetheless, BSI incidence remains high in the first 3 months of ART despite CPT. Further interventions to reduce BSI-associated mortality in the first 3 months of ART require urgent evaluation.

## Introduction

Bloodstream infection (BSI) is a common reason for adults to present to hospital in sub-Saharan Africa (SSA), particularly in countries with a high HIV-seroprevalence [1], [2], [3], [4]. Prior to the introduction of antiretroviral therapy (ART), sentinel surveillance at Queen Elizabeth Central Hospital (QECH), a large hospital in Malawi established a 70% adult in-hospital HIV prevalence and a 38% case fatality among individuals with culture confirmed BSI [5], [6]. A recent meta-analysis of BSI in Africa found that the most frequent bacterial causes of adult BSI in SSA were Nontyphoidal *Salmonellae* (NTS) and *Streptococcus pneumoniae*, and that except for *S.* Typhi BSI, HIV co-infection was a risk factor for any cause of BSI [7].

In Blantyre, Malawi, the predominant causes of BSI have been NTS and *S. pneumoniae* [1], [6]. Here there have been epidemics of BSI caused by multi-drug resistant (MDR) NTS starting in 1999 (*S*. Enteritidis) and 2002 (*S*. Typhimurium), however after these epidemics, NTS began to decline as a cause of BSI [8]. Between 85–90% of *S*. Typhimurium isolates at QECH have been resistant to amoxicillin, chloramphenicol and cotrimoxazole since the epidemic began. The MDR phenotype and the non-specific mode of presentation of iNTS in this setting [9] necessitated the introduction of the 3^rd^-generation cephalosporin, ceftriaxone for the empirical management of sepsis in 2004. A significant decline in invasive Pneumococcal disease, occurred between 2001 and 2010, prior to the roll-out of the 13-valent conjugate Pneumococcal vaccine in November 2011 [10]. Vaccine was not routinely available even in the private sector prior to this. There has been on vaccine trial of a 7-valent vaccine in adults [11] but this was too small to impact on these trends.”

Although 30% of Pneumococcal isolates are MDR, only 1–2% are resistant to a combination of crystalline-penicillin and chloramphenicol, in contrast 90% are resistant to cotrimoxazole. Up until 2009, extended-spectrum β-lactamase (ESBL) producing organisms had fortunately not been a major problem as carbapenems are not locally available. A recent study of paediatric malaria found a decline between 2001–3, but no change after 2005 despite the roll-out of malaria control interventions [12].

In 2000, the overall estimated urban HIV prevalence in Malawi was 18%, which remained stable at 17.4% in 2010 [13]. National roll-out of anti-retroviral therapy (ART) and cotrimoxazole preventive therapy (CPT) were commenced in 2004 and 2005 respectively [14]. Initially ART, (Triomune 30; consisting of stavudine, lamivudine and nevirapine) became available for patients presenting with WHO clinical stage III/IV disease and for those with a CD4 count of less than 200 cells/μL, which increased to 250 cells/μL in 2006. By the end of 2010, over 300,000 Malawians had started ART [15], 46% of the eligible population [14]. As of the end of June 2010, 95% of ART patients were on CPT and a cumulative total of 339,000 patients (pre-ART and ART) had been entered in CPT registers [15].

A fall in HIV-associated BSI has been described in industrial settings [16], but whether and how BSI has changed in the era of rapid ART and CPT roll-out in Africa has not been well described [17]. We therefore established an adult admissions cohort on the background of longstanding active routine surveillance for BSI at a large government hospital in Malawi to prospectively evaluate aetiology and outcome of suspected BSI episodes over a 1-year period. We have compared these findings with those from BSI studies performed in the same setting that pre-date ART and CPT roll out.

## Materials and Methods

### Setting

QECH has 1300 beds and is the largest government hospital in Malawi and provides free health care to Blantyre district (population approximately 1 million). QECH is the only free inpatient adult facility in Blantyre and most adults with severe febrile illness in urban Blantyre are therefore referred (∼10%) or self-present (∼90% - personal communication Dr M Nyirenda) to QECH. Up until 2011, when an Emergency Department opened, all adult patients presenting to QECH were triaged through a clinic assessment room. Patients are either discharged or admitted to a medical assessment unit by a clinical officer. Participants were recruited to the study at this stage. Individuals≥16 years are considered to be adults for the purposes of admission to the adult wards and therefore recruitment to the study. There was no substantial change in the provision of government health clinics outside of QECH from 1997–2010 and therefore no change in the referral pattern or admission numbers to QECH.

### Study Design: 2009/10 cohort

**Figure 1:**
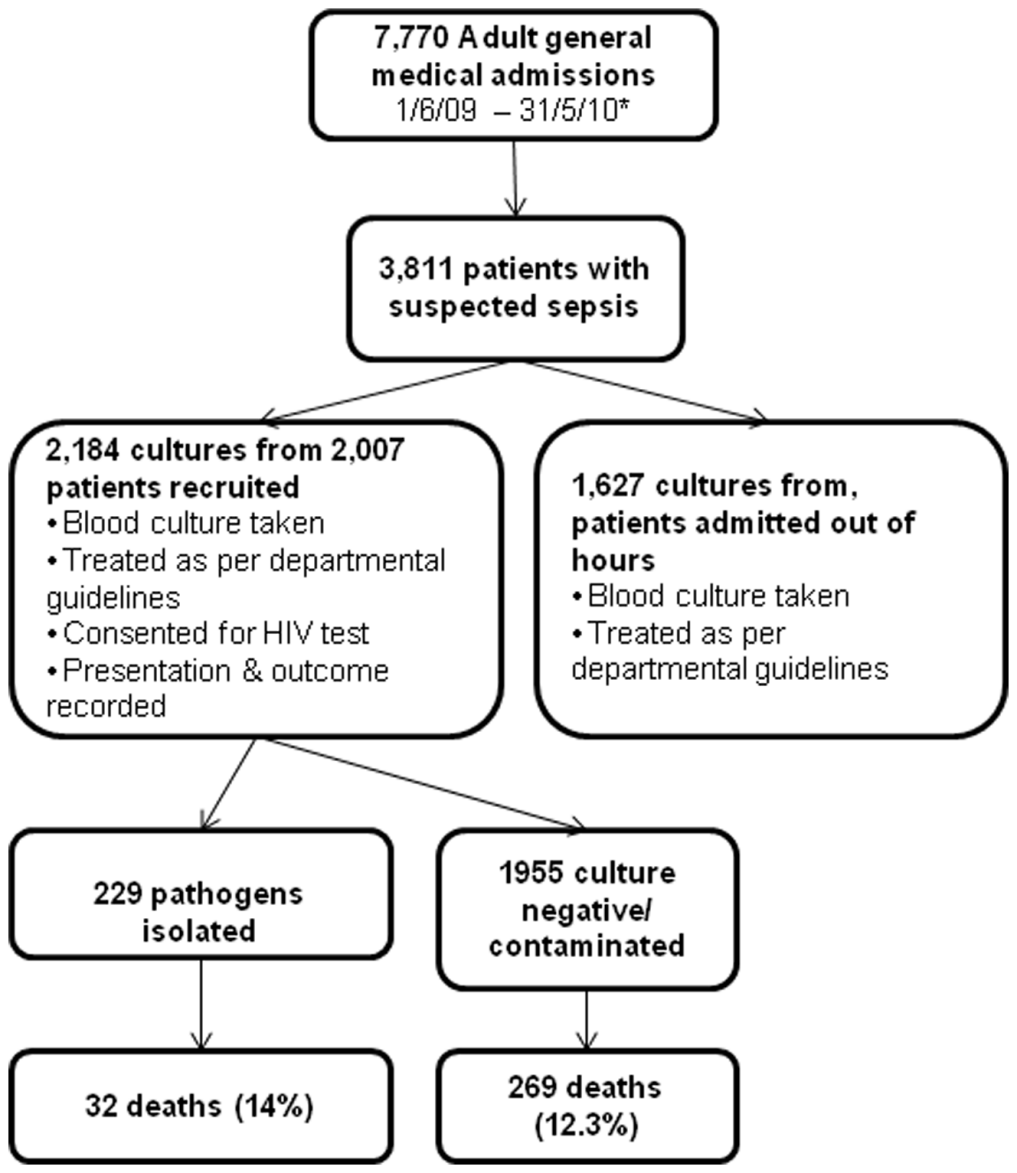
Summary of patient recruitment strategy and outcomes.

The Malawi Liverpool Wellcome Trust Clinical Research Programme (MLW) has conducted surveillance for BSI by blood culture since 1997 [6]. Aerobic blood cultures (7–10 mls blood) were routinely obtained for automated culture from all adult patients (age≥16) admitted to the medical assessment unit with an axillary temperature over 37.5°C or with clinical suspicion of sepsis (see figure 1). Blood cultures were routinely obtained before the administration of antimicrobials. Multiple blood cultures were not taken. Patients were consented and tested for HIV at this time (see File S1. Supplementary laboratory methods for protocol). Mycobacterial culture, serological and molecular diagnostics were unavailable.

In the present study, an enhanced clinical adult admissions cohort was established, and patients recruited from the existing admissions unit between 8am and 5pm on weekdays from 1^st^ June 2009 - 31st May 2010. Demographic, clinical, laboratory and in-hospital outcome data were collected on a standard case record form, including confirmed HIV-status. ART duration was calculated based on day of admission, adherence to ART and CPT was self-reported (other measures were not available) and HIV viral-load testing was not available. Standard laboratory methods, based on National Standard Methods (HCE, UK) and British Society of Antimicrobial Chemotherapy guidelines were used (see File S1. Supplementary laboratory methods) [18], [19]. All study patients were classified as suspected BSI and further classified as either culture-confirmed BSI (patients with a pathogen) or culture-negative (patients with a negative/contaminated culture (as defined previously[6])).”

Patients from outside the Blantyre area were excluded and patients whose blood cultures were obtained after hospital admission were not enrolled into this study, so nosocomial infection would not have been detected.

### Recruitment and management of historical, comparator cohort in 1997/1998

Comparison of microbiological diagnosis and clinical outcome was made to a similar cohort of febrile adults recruited in 1997/8 [6]. In the intervening 11 years, criteria blood culture have remained the same. Interventions to improve blood culture yield have been introduced, and blood culture has changed from a manual to an automated system (see File S1. Supplementary laboratory methods). The first generation of doctors graduated from the University of Malawi College of Medicine in 1992 and the numbers of graduating medical practitioners has increased from 10 per year to over 50 per year in the last five years. This has increased the numbers of interns and medical officers in the Department of Medicine at QECH ever since. Additionally, intravenous ceftriaxone (2g once daily) has replaced chloramphenicol and benzyl-penicillin in the empirical management of sepsis.

### Estimating minimum incidence of BSI for Blantyre

As QECH is the only hospital in the Blantyre district where treatment is freely available, it was possible to estimate a minimum incidence of BSI for the whole area. Study patients were categorized as HIV-uninfected; HIV-infected, but non-treated; ART-treated for ≤ 3months; and ART-treated for > 3 months.

The numerators were estimated from culture confirmed BSI cases observed in the study population. These were proportionately adjusted upwards by an adjustment factor based on the total number of culture confirmed adult BSIs (i.e. Including those from out of study hours) diagnosed at MLW during the study period. The adjustment factor was 1/(culture confirmed BSI recruited to study/total culture confirmed BSI at MLW during study period). We have assumed that the proportion of specific patient groups was the same in the study and non-study BSI suspects.

Denominators were estimated using the 2008 Malawi census projections and a published estimate of adult HIV-prevalence in urban Malawi [20] and stratified by age. All ART clinics are required to document numbers of patients starting and receiving ART, and those who default, transfer or die after starting ART and report these data to the Department of HIV and AIDS, Malawi Ministry of Health (MMH). A recent external data quality audit found that this system produced data of very high quality [14]. Person-years of observation were reduced in proportion to expected deaths during the observation period, recently estimated in Malawi at 37% during the first 3 months of ART and a further 8% in the following 9 months [14]. Deaths were assumed to be evenly distributed over the observation period.

### Statistical analyses

Statistical analyses were done using STATA for windows software (version SE/11; Stata corp, Texas USA). Where medians are used, interquartile range (IQR) is presented. Statistical testing was considered significant at the 5% significance level. Z-tests were used to compare differences in proportions between blood culture yield and mortality between the 2 cohorts. Univariate and multivariate logistic regression analysis were performed to identify potential factors associated with case-fatality and reported as odds ratios with 95% confidence intervals. Factors identified as significant by univariate analysis were included in the multivariate analysis. Incidence rates (IR) and incidence rate ratios (IRR) with their corresponding 95% confidence intervals were used to describe incidence of BSI in the 2009-10 cohort.

### Ethical approval

The study was given prospective ethical approval by the University of Malawi College of Medicine Research Ethics Committee (COMREC no. P.05/08/672). All patients or their relatives gave informed written consent prior to recruitment. Relatives were consented if patients were too sick to give consent as failure to recruit the sickest patients would have distorted the outcome of this study.

## Results

### General Characteristics of 2009/10 cohort

In total, MLW processed 3,811 blood cultures from adult patients between 1^st^ June 2009 and 31^st^ May 2010, and 449 pathogen positive blood cultures were detected. In this study, a total of 2184 cultures (1 per patient per visit) were collected including 177 blood-cultures for repeat admissions (see figure 1). Thus a total of 2,007 adults with clinically suspected BSI were recruited between July 2009 and June 2010. 908/2,007 (45%) were male. Only 126 patients (6%) had been admitted to hospital in the previous year (table 1). 1902/2007 (95%) patients consented to HIV testing and 1717 (90%) were HIV-infected. 623/1717 (36%) were taking CPT and 485/1717 (28%) patients were taking ART at presentation. 212/485 (44%) were taking ART for less than 3 months. 357/2056 (17%) of patients had been prescribed antimicrobials before presentation, although they were no less likely to yield a pathogen from blood culture (OR .96 [95% CI: 0.6–1.4]).

**Table 1:** Basic description of clinical and laboratory data for the 2009/2010 cohort.

	Number	%
**Male Gender**	908/2,007	45.2
**Age Mean(sd)**	34.4 (12.6)	
**Clinical features suggestive of HIV at presentation** *	1169/2,007	58.2
**Total HIV infected**	1717/1902	90.0
**Taking ART**	485/1717	28.2
0–3 months	212/485	43.7
>3 months	273/485	56.3
**Taking cotrimoxazole prophylactic therapy**	623/1717	36.3
**Clinically suspected focus of infection**		
Respiratory	376/1228	30.6
Central Nervous Syndrome	78/1228	6.4
Gastrointestinal	22/1228	1.8
Non focal sepsis	669/1228	54.4
Other	83/1228	6.8
**Median Haemoglobin (g/dl, IQR)**	10.0 (7.6–12.0)	
**Median CD4 (x10^9^ cells/l, IQR)**	100 (33–199)	

*At least one of Kaposi's sarcoma, oral candidiasis, wasting, generalized lymphadenopathy or herpes-zoster.

The syndromic diagnoses were notably similar to those reported in a similar study conducted in 2000 [1], with lower respiratory tract infection (376 cases) and non-focal sepsis (669 cases) remaining the most common. In 1686/2,007 (84%) of episodes, intravenous (IV) ceftriaxone was commenced at presentation.

The median Hb was 10.0 g/dl (IQR 7.6–12.0). Of the 367/1717 (21%) patients with a documented CD4 count, 275 (75%) had a count less than 200×10^9^ cells/l (median 100×10^9^ cells/l [IQR 33–199]).

Among the 1,400/2,007 (70%) study patients tested for malaria, 272 (15%) were slide positive for *P. falciparum*, of whom 14 (6%) were also bacteraemic.

### Causes of BSI 2009/10 and comparison to historical cohort

Of 2184 episodes of suspected BSI in the 2009/10 cohort, 229 (10%) yielded a pathogenic organism, a significant decrease when compared to 449/2789 (16%; p<0.001) in the 1997/8 study (table 2). A further 157 cultures (7%) were contaminated. Amongst the non-study blood cultures, 220/1627 (14%) grew a significant isolate and 51% (229/449) of the total yield of pathogens during the study period, were from study participants. One patient had 2 pathogens isolated from a single blood-culture.

**Table 2:** Comparison of bacterial pathogens isolated from blood culture in 1997/98 and 2009/10.

	1997/8 [6]			2009/10			Change in % of total cultures: 1998–2010 (%)*	Z test P value
	Number	% of significant bloodstream isolates	% of total cultures	Number	% of significant bloodstream isolates	% of total cultures		
**Gram negatives**								
Total NTS†	164	37.9	5.9	84	36.7	3.8	35.6	<0.001
*S*. Enteritidis	26	6.0	0.9	10	4.4	0.5	44.4	0.10
*S*. sp.	10	2.3	0.4	7	3.1	0.3	25.0	0.56
*S*. Typhimurium	128	29.6	4.6	67	29.3	3.1	32.6	0.004
*S*. Typhi	12	2.8	0.4	9	3.9	0.4	0.0	1.0
*E. coli*	43	9.9	1.5	35	15.3	1.6	+6.7	1.0
*Klebsiella* sp.	19	4.4	0.7	5	2.2	0.2	71.4	0.02
Other Gram Negative Bacilli	37	8.5	1.3	12	5.2	0.5	61.5	0.004
**Gram positives**								
*S. pneumoniae*	136	31.4	4.9	56	24.5	2.6	46.9	<0.001
*S. aureus*	6	1.4	0.2	7	3.1	0.3	+50.0	0.48
Other Gram Positive pathogens	16	3.7	0.6	5	2.2	0.2	66.7	<0.001
Pathogenic Yeasts	8	1.8	0.3	16	7.0	0.7	+133.3	0.042
**Total pathogens**	433	100	15.5	229	100	10.4	32.9	<0.001
**Total blood cultures**	2789			2184				

† NTS are evaluated both in total and broken down by serotype. *All figures are % fall, unless indicated by “+”.

The most common culture-confirmed cause of BSI remained NTS (84/229 [37%]). Between the periods 1997/8 and 2009/10 the proportion of all samples yielding NTS declined significantly from 6% to 4% (p = 0.0008), reflecting the overall reduction in BSI. In 1997/8 100% of NTS were susceptible to chloramphenicol. In the present study, 66/84 (79%) of NTS isolates were MDR (resistant to amoxicillin, chloramphenicol and cotrimoxazole) and 73/84 (87%) were resistant to cotrimoxazole. Cephalosporin and ciprofloxacin resistance were not observed.

*S. pneumoniae* was the second most frequent BSI isolate (56/229 isolates [25%]), however between 1997/8 and 2009/10 the proportion of all cultures yielding *S. pneumoniae* fell from 5% to 3% (p<0.0001). The proportion of *S. pneumoniae* resistant to penicillin by disc testing was 12/54 (22%), an increase from 11% in 1997/8, whilst 54/57 (95%) were resistant to cotrimoxazole [10]. There was 1 cephalosporin resistant strain of *S. pneumoniae* in 2009/10.

There were 35/229 (15%) *E. coli* isolates, which did not represent a significant change from 1997/8 (43/433 [10%] isolates, p = 0.95), whereas *Klebsiella spp* were identified in only 6/229 (2%) isolates compared with 19/433 (4%) in 1997/8 (p = 0.02). 34/35 (97%) *E. coli* and 5/6 (83%) *Klebsiella* were resistant to cotrimoxazole.

Broader spectrum antimicrobials, ciprofloxacin (2002) and ceftriaxone (2004), were introduced at QECH in between these studies. Despite this, Methicillin-resistant *Staphylococcus aureus* (MRSA, 1 isolate only) and extended spectrum beta-lactamase (ESBL) producing Enterobacteriaceae, 4 *E. coli* (also resistant to ciprofloxacin), were isolated rarely. There were a further 3 ciprofloxacin resistant isolates in the study (1 *E. coli*, 1 *K. pneumoniae* and 1 *C. freundii*.

In addition to the bacterial pathogens, *C. neoformans* was isolated in 15/229 (7%) cases of BSI, 0.7% of all cultures and *Candida albicans* was isolated once. This represents a rise since 1997/8 when *C. neoformans* was isolated in 8/433 (2%) cultures whilst *C. albicans* was not isolated at all in the previous study.

### Clinical outcomes

Mortality among all 2184 episodes of suspected BSI declined from 18% in 1997 to 12% in 2010 (p-value<0.001)(table3). Case fatality was14% in the 229 episodes of culture-confirmed BSI in 2009/10, significantly decreased from 39% in 1997 (p<0.001) (table 3). In contrast, the fall in mortality in culture-negative patients was not significantly different (331/2356 [14%] in 1998 to 240/1971 [12%] in 2010, p = 0.066); most of the overall reduction in mortality observed was due to improvements in outcome from culture-confirmed BSI.

**Table 3:** Comparison of case fatality rates 1997/98 & 2009/10.

	1997/8 [6]		2009/10		
	n		n	%	z- test (p-value)
All-cause mortality	502/2789	18.0	269/2184	12.3	<0.001
Blood culture-confirmed BSI	171/433	39.5	32/229	14.0	<0.001
Nontyphoidal Salmonellae	54/164	32.9	9/84	10.8	<0.001
*S. pneumoniae*	49/136	36.0	4/56	8.9	<0.001
Blood-culture negative/contaminated	331/2356	14.0	237/1955	12.1	0.066

In the present cohort, male sex (OR 1.7: 95% CI 1.3–2.2), anemia (OR 2.4: 95% CI 1.5–3.8) and HIV status (OR 2.3: 95% CI1.3–4.1) were identified as risk factors for increased mortality among febrile admissions by univariate analysis (table 4). Neither a positive blood culture, nor any of the common bloodstream pathogens were associated with a significantly increased risk of death (table 4). Following multivariate analysis, anemia (OR 3.0 [95% CI: 1.7–5.3]) and male gender (OR 1.7 [95% CI: 1.2–2.5]) remained independent risk factors for mortality.

**Table 4:** Factors associated with death among patients admitted with clinically suspected BSI in 2009/10.

Variable	All patients				HIV-infected patients			
	Univariate Analysis		Multivariate Analysis		Univariate Analysis		Multivariate Analysis	
	OR	95% CI	Adjusted OR	95% CI	OR	95% CI	Adjusted OR	95% CI
**Male Gender**	1.7	(1.3–2.2)	2.0	(1.4–2.9)	1.9	(1.4–2.4)	2.0	(1.4–3.0)
**HIV infection**	2.3	(1.3–4.0)	1.4	(0.7–2.9)	Unity			
**Anaemia (WHO criteria** [33] **)**								
Mild anaemia	1.4	(0.8–2.3)	1.6	(0.9–2.7)	1.7	(1.0–2.9)	1.6	(0.9–2.8)
Moderate anaemia	1.5	(0.9–2.3)	1.6	(1.0–2.7)	1.7	(1.0–2.7)	1.6	(1.0–2.8)
Severe anaemia	4.3	(2.8–6.7)	4.5	(2.7–7.6)	4.7	(2.9–7.7)	4.5	(2.6–7.7)
**Blood stream infection**								
Malaria	0.6	(0.4–1.0)	0.8	(0.5–1.5)	0.6	(0.4–1.0)	0.9	(0.5–1.6)
iNTS	0.8	(0.4–1.6)	0.6	(0.2–1.4)	0.7	(0.4–1.4)	0.4	(0.1–1.2)
Other Enterobacteriaceae	1.8	(0.9–3.4)	1.1	(0.4–3.0)	1.6	(0.8–3.1)	1.1	(0.4–3.2)
*S. pneumoniae*	0.5	(0.2–1.4)	0.4	(0.1–1.8)	0.5	(0.2–1.4)	0.2	(0.0–1.5)
**Therapy (if HIV infected) vs HIV negative**								
CPT					1.2	(0.9–1.6)	1.0	(0.6–1.8)
No ART					0.5	(0.1–5.0)	1.2	(0.6–2.5)
ART<3 months					0.6	(0.1–5.7)	1.4	(0.5–3.9)
ART≥3 months					0.5	(0.0–4.6)	1.2	(0.5–3.3)

HIV-infected patients were analyzed separately, enabling ART status and cotrimoxazole to be included in the model (table 4). In this analysis, male gender (OR 1.7 [95% CI: 1.3–2.2]) and severe anemia (OR 4.3 [95% CI: 2.8–6.7]) were again significant risk factors for mortality.

### Co-trimoxazole preventive therapy

There was no significant difference in mortality due to BSI between HIV-infected patients on CPT and those who were not (OR 1.87 [95% CI: 0.88, 3.99]). HIV-infected patients with suspected BSI and taking CPT were no less likely to have culture-confirmed BSI than those who were not (OR 0.86, 95% CI 0.61–1.20). Patients taking CPT were significantly more likely to have BSI from a cotrimoxazole resistant organism (OR 7.0 [95% CI 1.6–31.2]), but significantly less likely to be slide-positive for malaria parasites than those who were not (OR 0.35 [95% CI 0.24,0.53]).

### Impact of ART on presentation with and mortality from suspected BSI

The minimum incidence of culture-confirmed BSI was estimated at 0.06/1000 person-years in the HIV-uninfected adult population. Taking into account all culture-confirmed BSI diagnosed at MLW during the study period, including those from out of study hours this figure was adjusted upwards by a factor of 1/0.51 to 0.12/1000 to reflect the total BSI burden (table 5).

**Table 5:** Minimum incidence of BSI in the Blantyre district.

HIV Status	Age group (years)	Culture confirmed BSI		Estimated population in Blantyre district 2009/10	Estimated person years of observation	Minimum incidence BSI/1000 patient yrs		Incidence rate ratio relative to HIV uninfected
						Crude	Adjusted*	(95% CI)
**HIV uninfected**	all	14/171	8.2	475,214	463,334	0.06	0.12	
**HIV infected, no ART**	all	148/1180	12.5	65,763	60,831	4.86	9.72	80.3 (46.4,138.9)
**HIV + ART <3/12**	all	30/212	14.2	9,124	1,718	34.92	69.84	568.0 (301.7,1069.3)
**HIV + ART >3/12**	all	24/273	8.8	27,298	26,206	1.84	3.68	30.3 (15.7,58.5)
**Total**				587,270	552,088			

*The adjustment factor was 1/(culture confirmed BSI recruited to study/total culture confirmed BSI at MLW during study period).

Adjusted minimum incidence among the HIV-infected adult population not on ART was estimated at 9.6 and at 68.4 in those on ART<3 months (4.3 amongst HIV-infected patients overall). Incidence declined to 1.8 in those on ART>3 months (table 5). Rate of proven BSI was significantly lower amongst patients who had been on ART for >3 months than those either not on ART (p = <0.0001) or on ART<3 months (p = <0.0001).

We calculated estimated incidence rate ratios (e-IRR) of BSI using HIV-uninfected patients as the comparator group and stratified these results by age. HIV-infected adults not on treatment had a crude e-IRR of BSI of 80 (95% CI: 46,139), for those on ART for less than 3 months the e-IRR was 568 (95% CI: 302,1069), while for those on ART>3 months it was 30 (95% CI: 16,59).

Amongst patients on ART for<3 months, there were 30 BSIs, including 13 iNTS, 5 *S. pneumoniae*, 8 other bacterial infections and 4 *C. neoformans*. The relative frequency of these pathogens was similar to that seen at other stages of HIV treatment and no single agent was over represented during immune-reconstitution. The median time from commencing ART to presenting with BSI was 33 days (IQR 9, 66).

The minimum case fatality rate (CFR) from suspected sepsis per 1,000 adults per year in Blantyre based on study participants was 0.03 amongst HIV-uninfected patients, 2.84 in HIV-infected patients not on therapy, 18.04 in patients in the first 3 months of ART and 1.37 in patients on ART for greater than 3 months. If we adjust the estimate to include the number of suspected sepsis cases presenting out of study hours, whilst adjusting for repeat cultures, we estimate a minimum CFR of 0.05 in HIV-uninfected patients, 4.8 in HIV-infected patients not on treatment, 30.7 in those in treatment for less than 3 months and 3.3 in those on treatment for more than 3 months. We assume case fatality is similar by clinical group in the out of hours and study hours recruitment periods. Mortality risk ratios when compared to HIV-uninfected patients are 93.9 (95% CI: 54.4–161.8) for untreated HIV patients, 586.6 (95% CI: 312.6–1100.7) for those on treatment for less than 3 months and 45 (95% CI:24.5–84.2) for those on treatment more than 3 months.

## Discussion

A highly effective public health approach has been used to roll-out ART in Malawi, a high HIV-prevalence, resource-poor country. Benefit in terms of a reduction of mortality has previously been demonstrated for ART at population level [21],[22]. Our study suggests that this benefit is likely in part to have derived from the prevention of BSI in patients on longer-term ART. This is consistent with data from verbal autopsy results from Northern Malawi that indicated that AIDS related infections were the most common causes of mortality prevented with progressive ART scale up [23].

There have been striking improvements in case fatality between 1997/8 and 2009/10 and it is likely that the improved recognition of BSI, better clinical care and greater availability of broad spectrum antibiotics (i.e. ceftriaxone) that have occurred since 1998 have all contributed to the observed improvement in survival. In addition to improved in-patient care, the marked reduction in the eIRR of BSI and risk of death from suspected sepsis amongst patients on ART≥3 months compared with those not taking ART or in the early stages of immune reconstitution, is consistent with ART as one of the primary drivers of these changes. The protective effect of successful HIV-treatment might well have been under-estimated by his study, however we were not able to perform viral load assays to distinguish patients with fully suppressed HIV-viral loads from those with evidence of HIV-treatment failure. Although reports from Malawi suggest that significant primary ART resistance is, as yet uncommon [24], there are few data on HIV-treatment failure in this setting. In Blantyre in 2014, there is expanded access CD4 count testing, an improved prevention of mother to child transmission programme and progress has been made towards starting ART at CD4 counts of<350 cells/μl using tenofovir, lamivudine and efavirenz based therapy, all of which might be expected to reduce BSI even further.

CPT has been shown to be effective in reducing mortality in Zambian children and Ugandan adults [25], [26]. In our study, CPT use was not associated with an overall reduction in culture-confirmed BSI or decreased case fatality, but only with protection from malaria (as previously reported) [27]. Whilst this study was not designed to evaluate the efficacy of CPT, we found nearly universal resistance to cotrimoxazole amongst key pathogens, which could render CPT less effective against *S. pneumoniae* and NTS. Pneumococcal carriage studies of HIV-infected adults in Malawi have shown high rates of colonisation despite CPT [28] and the efficacy of CPT against both carriage and invasive disease should be further investigated. Given the higher incidence of BSI during the initial induction of ART and the persistently high mortality during this period [14], we suggest that the utility of cotrimoxazole should be re-evaluated and alternative prophylactic agents tested.

It is noteworthy that in this high HIV-prevalence population, 88% of deaths occurred amongst blood culture negative patients. Part of the mortality may have been due to BSI masked by prior antibiotics, BSI caused by bacteria which are difficult to culture (i.e. of genus Brucella, Leptospira, or Rickettsia), or other pathogens which are cannot be diagnosed by bacteria culture (i.e. viruses) [29]. Blood culture is an imperfect technology that is more sensitive for some pathogens (i.e. Enterobacteriaceae) than others (*S. pneumoniae*). Also, we and others have shown that TB is a common cause of BSI with high mortality in this setting particularly in the very immunocompromised and that unrecognised TB explains some of these deaths [30], [31]. Lastly, not all fatal infections cause BSI, for example pneumococcal pneumonia may cause death without BSI. Formal evaluation of novel diagnostics to more rapidly identify and treat smear negative TB and TB BSI is required [32].

### Limitations

The measured incidence rates of BSI have several potential sources of error but are likely to underestimate BSI and therefore represent reasonable minimum estimates of the disease burden. The numerator data were derived from patients that presented and were recruited in normal working hours, however blood cultures taken out of hours were modestly, but significantly more likely to be culture positive (OR 1.33 [95% CI: 1.10–1.63]). Our denominator data are derived from Malawian census, Malawi DHS report (2010) and Malawi MoH data, all of which are audited and which are believed to be robust and accurate [14]. Greater scope for error is likely to come from measurement of cases, as individuals accessing care at other health centres or who died before reaching QECH could not be sampled. Comparisons between study periods may have been confounded by unmeasured changes in healthcare utilization and delivery.

There are very few laboratories conducting robust long-term surveillance for BSI in large urban populations in sub-Saharan Africa and therefore the data presented are restricted to a single centre in Malawi. Evidence from earlier BSI studies in Malawi and other populations throughout eastern and southern Africa would suggest similarities between these sites and therefore these data are likely to be generalizable to other African settings [6], [7]. Our laboratory surveillance was limited by lack of mycobacterial, serological and molecular diagnostics. Also, funding was not available for all patients to have a CD4 count performed and this test was restricted according to hospital policy to patients who were expected to survive admission and who did not meet the criteria for ART based on WHO stage, and therefore only 367 patients had a CD4 count.Follow-up in the present study was confined to in-hospital outcomes and therefore it is possible that there was an unrecognised high out-of-hospital early mortality following discharge [30]. However, the improved survival seen in this study is consistent with nationally collected ART outcome data [14].

Whilst criteria for admission and blood culture did not change in the interval between 1997/8 and 2009/10, baseline characteristics were not recorded in the earlier study. Although the recruitment criteria between studies did not change, the baseline characteristics of the cohorts cannot be matched.

## Conclusions

Our data suggest that following the successful roll-out of ART, the incidence of BSI has fallen and clinical outcomes from BSI have improved significantly in a high HIV-prevalence African setting. Nonetheless, BSI incidence remains high in the first 3 months of ART despite cotrimoxazole prophylaxis. To ensure the continued success of ART programmes throughout sub-Saharan Africa, approaches to the intensification of BSI prophylaxis during the initiation of ART need to be explored and it is hoped that the results of the REALITY study (ISRCTN43622374) may offer a solution to the high early mortality we report. In particular, the effective long-term control of *S. pneumoniae* and NTS infection, whether through vaccination or improved socio-economic status, including access to clean water and sanitation require further evaluation. Additional interventions to reduce the considerable BSI-associated early mortality in the first 3 months of ART require urgent evaluation.

## Supporting Information

File S1 **Supplementary laboratory methods: A detailed description of the diagnostic laboratory methods employed, including microbiology, haematology and HIV diagnostic kits used.**
(DOCX)
