# Supplementary material for: A Reduction in Adult Blood Stream Infection and Case Fatality at a Large African Hospital following Antiretroviral Therapy Roll-Out
Source: PLoS One. 2014 Mar 18;9(3):e92226. doi: 10.1371/journal.pone.0092226 (PMC3958486; doi:10.1371/journal.pone.0092226)
Supplement: File S1 — Supplementary laboratory methods: A detailed description of the diagnostic laboratory methods employed, including microbiology, haematology and HIV diagnostic kits used. (DOCX) [file pone.0092226.s001.docx]

**Laboratory methods:**

Automated blood culture was undertaken using a standard aerobic bottle (BacT/Alert BioMerieux) by MLW. Blood culture volumes and contamination rates are audited and the impact of MLW’s blood culture service described [[1](#_ENREF_1)].

All isolates were identified using standard diagnostic techniques [[2](#_ENREF_2)]. Coagulase-negative *Staphylococci*, bacillus spp, diptheroids and alpha-haemolytic *Streptococci* other than *S. pneumoniae* (when there was no clinical suspicion of endocarditis) were considered as contaminants. Anaerobic and mycobacterial culture facilities were not available.

Antimicrobial susceptibility testing was performed by disc diffusion according to British Society of Antimicrobial Chemotherapy standards, however no minimum inhibitory concentration testing was available [[3](#_ENREF_3)]. Penicillin resistance in *S. pneumoniae* was defined as a zone size of ≤19mm against a 1µg oxacillin disc on iso-sensitest agar supplemented with 5% sheep blood incubated overnight in 5% CO_2_. External Quality Assurance was monitored by subscription to the appropriate United Kingdom National External Quality Assessment Service (UK NEQAS) schemes. The antimicrobials which each organism was tested against during the study period are listed in Table 1.

A thick blood film was examined for Plasmodium parasites. HIV testing was performed according to the national HIV rapid antibody testing protocol, using Determine^TM^ HIV-1/2 tests as the first test in a serial testing algorithm. All positive test results were confirmed by Uni-Gold^TM^. Full blood count was tested by one of three machines (SYSMEX KX-21N, ABX MICROS 60, Beckman Coulter Ac-T 5diff CP). Haemoglobin (Hb) levels were classified following WHO recommendations (anaemia: <13.0 g/dl in men or <12.0 g/dl in women; severe anaemia: Hb <8.0g/dl in men and women)[[4](#_ENREF_4)]. CD4 count was performed on a Beckman Coulter EPICS, but not routinely checked during the study period.

Table 1: Table of antimicrobial discs used in susceptibility testing. Shaded box indicates susceptibility testing is performed

|  | Enterobacteriaceae | Pseudomonas | S. pneumoniae | S. aureus | Other Streptococci |
| --- | --- | --- | --- | --- | --- |
| Amoxicillin |  |  |  |  |  |
| Chloramphenicol |  |  |  |  |  |
| Cotrimoxazole |  |  |  |  |  |
| Gentamicin |  |  |  |  |  |
| Ciprofloxacin |  |  |  |  |  |
| Ceftriaxone | * |  |  |  |  |
| Oxacillin |  |  |  |  |  |
| Tetracycline |  |  |  |  |  |
| Methicillin |  |  |  |  |  |
| Erythromycin |  |  |  |  |  |
| Penicillin |  |  |  |  |  |

* If resistant, then ESBL-producing status confirmed as per BSAC guidelines [[3](#_ENREF_3)]

**Laboratory methods: 1997/8**

The methods used in this study have previously been described [[5](#_ENREF_5)]. In brief, there was no dedicated team of blood-culture nurses at this time and culture was manual, not automated. Blood was inoculated into brain-heart infusion broth and sub-cultured after 1, 2 and 7 days of culture at 37^o^C in air. This approach is likely to be of lower sensitivity than the 2009/10 methodology, especially for the culture of S. pneumoniae[[1](#_ENREF_1)].

1. Mtunthama N, Gordon SB, Kusimbwe T, Zijlstra EE, Molyneux ME, et al. (2008) Blood culture collection technique and pneumococcal surveillance in Malawi during the four year period 2003-2006: an observational study. BMC Infect Dis 8: 137.

2. Barrow G FR (1993) Cowan and Steele’s manual for the identification of medical bacteria. . Cambridge, UK: Cambridge University Press.

3. BSAC (2009) Methods for Antimicrobial Susceptibility Testing Version 8, January 2009 British Society for Antimicrobial Chemotherapy.

4. WHO (2011) Haemoglobin concentrations for the diagnosis of anaemia and assemssment of severity Vitamin and Mineral Nutrition Information System WHO/NMH/MNM/11.1.

5. Gordon MA, Walsh AL, Chaponda M, Soko D, Mbvwinji M, et al. (2001) Bacteraemia and mortality among adult medical admissions in Malawi--predominance of non-typhi salmonellae and Streptococcus pneumoniae. J Infect 42: 44-49.
